# Database screening of herbal monomers regulating autophagy by constructing a "disease-gene-drug" network

**DOI:** 10.1186/1472-6882-14-466

**Published:** 2014-12-04

**Authors:** Chenjun Hao, Zhengpeng Yang, Bo Gao, Ming Lu, Xianzhi Meng, Xin Qiao, Dongbo Xue, Weihui Zhang

**Affiliations:** Department of General Surgery, the First Affiliated Hospital of Harbin Medical University, Harbin, China; Department of Surgery, David Geffen School of Medicine, University of California at Los Angeles, Los Angeles, CA USA

**Keywords:** Herbal monomer, Autophagy, Genomics, Bioinformatics

## Abstract

**Background:**

Studies suggest an important role of autophagy as a target for cancer therapy. We constructed a "disease-gene-drug" network using the modular approach of bioinformatics and screened herbal monomers demonstrating functions related to autophagy regulation.

**Methods:**

Based on the microarray results of the gene expression omnibus (GEO) database (GSE2435 and GSE31040, starvation-induced autophagy model), we used the human protein reference database (HPRD) to obtain the protein-protein interaction (PPI) network. In addition, we used the CFinder software to identify several functional modules, performed gene ontology-biological process (GO-BP) functional enrichment analysis using the DAVID software, constructed a herbal monomer-module gene regulatory network using literature search and the Cytoscape software, and then analyzed the relationships between autophagy, genes, and herbal monomers.

**Results:**

We screened 544 differentially expressed genes related to autophagy, 375 pairs of differentially expressed genes, and 7 gene modules, wherein the functions of module 3 (composed of 7 genes) were enriched in "cell death". Using the constructed herbal monomer-module gene regulatory network, we found that 30 herbal monomers can simultaneously regulate these 7 genes, indicating a potential regulatory role in autophagy.

**Conclusions:**

Database screening using the disease-gene-drug network can provide new strategies and ideas for the application of herbal medicines in cancer therapy.

## Background

For several thousands of years, natural products have been used as medicines. Indeed, since ancient times, humans have searched for naturally occurring substances to alleviate their sufferings. Before the invention of synthetic drugs, natural drugs were the only choice for treating diseases. Even today, many of the world's populations still use natural medicine to treat diseases, including traditional Chinese medicine. With the development of science and technology, in-depth research has been conducted on ancient drugs using modern approaches to isolate effective monomers from complex natural mixtures, and remarkable achievements have been made. Natural products are important sources of new anti-cancer drugs, as more than 60% of anti-cancer drugs have been derived from natural products. Recently, a significant number of anti-cancer drugs derived from natural products, especially drugs of plant origins such as rhoeadine, podophyllotoxin, paclitaxel and camptothecin, have achieved great success in clinical application [1, 2].

Autophagy is an evolutionarily conserved process involved in homeostasis by degrading organelles and proteins to maintain biosynthesis during nutrient deprivation or metabolic stress [3]. This process is important for the elimination of damaged organelles and proteins with long half-lives. Defect in autophagy is associated with tumorigenesis via metabolic stress and DNA damage [4]. Loss of *Beclin-1*, an essential autophagy gene, is present in 40-75% of solid tumors, suggesting a tumor suppresion role of autophagy [4]. Although autophagy has been identified as a type of programmed cell death [5–7], autophagy is also a pro-survival process that enables tumor cells to survive adverse conditions such as hypoxia and chemotherapy [3, 8]. Autophagy is a pathway that is distinct from apoptosis, although some crosstalk exists between them [9, 10]. Therefore, because autophagy plays a complex and very important role in the pathogenesis and development of tumors, this process has become an important anti-cancer therapy target. As a result, approaches to effectively regulate autophagy are increasingly becoming the subject of active research.

In order to systematically identify herbal monomers for potential application in tumor treatment with regards to their ability to regulate autophagy, this study used the approaches of modular bioinformatics analysis and network pharmacology. Theoretically, if the inhibition of a certain gene can affect the pathogenesis and development of a disease, drugs regulating this gene might be effective for its treatment. In addition, it has been noted that disease occurrence is the result of changes in the transcription of multiple genes or gene groups in the human genome. Therefore, drug treatment should also target the transcription of these genes or gene groups in order to be more effective. Integrated analysis using "disease-gene" and “gene-drug" networks facilitate the identification of herbal monomers regulating autophagy, providing novel uses for old drugs.

## Methods

### Microarray data processing

Two series of Earle’s balanced salt solution (EBSS) starvation-induced autophagy model in a human lymphoblastoid cell line were selected in this study, including GSE2435 (http://www.ncbi.nlm.nih.gov/geo/query/acc.cgi?acc=GSE2345) [11] and GSE31040 (http://www.ncbi.nlm.nih.gov/geo/query/acc.cgi?acc) [12], which can be obtained from the GEO (Gene Expression Omnibus) database. The differentially expressed genes were detected based on the HG-U133-Plus-2 Affymetrix Human Genome U133 Plus 2.0 Array. Each experiment was repeated three times.

The microarray experiments performed in this study included the controls and the autophagy conditions in human cells, with three samples for each condition. We downloaded the microarray CEL data compression package from the GEO supplementary file and extracted the package to a folder for later use. We also downloaded the raw data in TXT format. We used Bioconductor and R 2.10.1 to normalize the downloaded data. A robust multi-array average (RMA) algorithm was applied to calculate the expression levels, and a multiscale adaptive search (MAS) algorithm was used to calculate the detection calls. Each group of samples with no less than 2 detection calls was retained and called Present (P), in order to filter low-expression probe sets. The HUGO Gene Nomenclature Committee (HGNC) gene names for human genes were used to unify the gene names. We then used the VLOOKUP function to replace the probe numbers in the original data and established the expression data sheet containing the gene names and the corresponding samples. We used the LIMMA differentially expressed gene screen algorithm to screen genes either upregulated or downregulated in the autophagy condition compared with the control condition, and the fold of change, P-value and false discovery rate (FDR) were calculated. P-values <0.5 or >2 indicated differentially expressed genes.

The intersection between GSE2435 and GSE31040 was used to screen the differentially expressed genes shared by the two microarrays.

### Generation of differentially expressed gene modules and functional analysis of the modules

We obtained the protein-protein interaction (PPI) network from the Human Protein Reference Database (HPRD). This network contains 36,874 edges and 9,453 nodes [13]. The differentially expressed genes were mapped to the PPI network, and pairs in which the two interacting proteins were both differentially expressed were retained. Subsequently, sub-networks were generated.

We used the CFinder software (http://cfinder.org/) to identify functional modules in the sub-networks. CFinder [14] was used for finding and visualizing concealed groups and modules in networks, based on the clique percolation method (CPM). CFinder can find the full join set of a specified size in the network and construct larger node groups through the shared nodes and edges in the full join set.

We used the Database for Annotation, Visualization and Integrated Discovery (DAVID) software (v6.7) for gene ontology-biological process (GO-BP) analysis of the screened modules. We opened the DAVID database (http://david.abcc.ncifcrf.gov/), submitted the gene groups proposed for further analysis, checked the entire human genome as the background genes, selected "Functional Annotation Tool" as the analytical tool, and then obtained the GO-BP enrichment analysis results (P-value was 0.05).

A file of the interesting genes and their corresponding GO-BP terms was generated. The file was imported to Cytoscape (version 2.6.3) [15], the interaction type was "default interaction", and the "layout", "cytoscape layout" and "spring embedded" options were selected. At last, a network illustrating the functions of interesting genes was constructed.

### Construction of the herbal monomer-module gene regulatory networks

Based on the herbal monomer database (http://www.sepu.net/html/article), we established a file of herbal monomers including 30 monomers mightly related to autophagy, entered the file of differentially expressed module genes and the file of herbal monomers into the ActivePerl 5.16.2 software, and extracted a literature set from the PUBMED database that was related to "each differentially expressed module gene" and "each herbal monomer". We also conducted preliminary grammar and syntax analyses of the target literature set, analyzed the potential conceptual relationship in the literature, and identified the corresponding herbal monomers regulating target genes, followed by manual screening. We then drew the herbal monomer-module gene regulatory network diagram using the Cytoscape software (version 2.6.3).

## Results

### Detection of differentially expressed genes after autophagy induction

GSE2435 detected a total of 13,234 genes. Taking a P-value <0.05 as the significance level of differentially expressed genes, we screened a total of 2783 differentially expressed genes. GSE31040 detected a total of 14,442. Taking a P-value <0.05 as the significance level for differentially expressed genes, we screened a total of 2,369 differentially expressed genes. There were a total of 544 differentially expressed genes identified for the intersection of GSE2435 and GSE31040.

### Modular analysis results of autophagy-related genes

**Figure 1:**
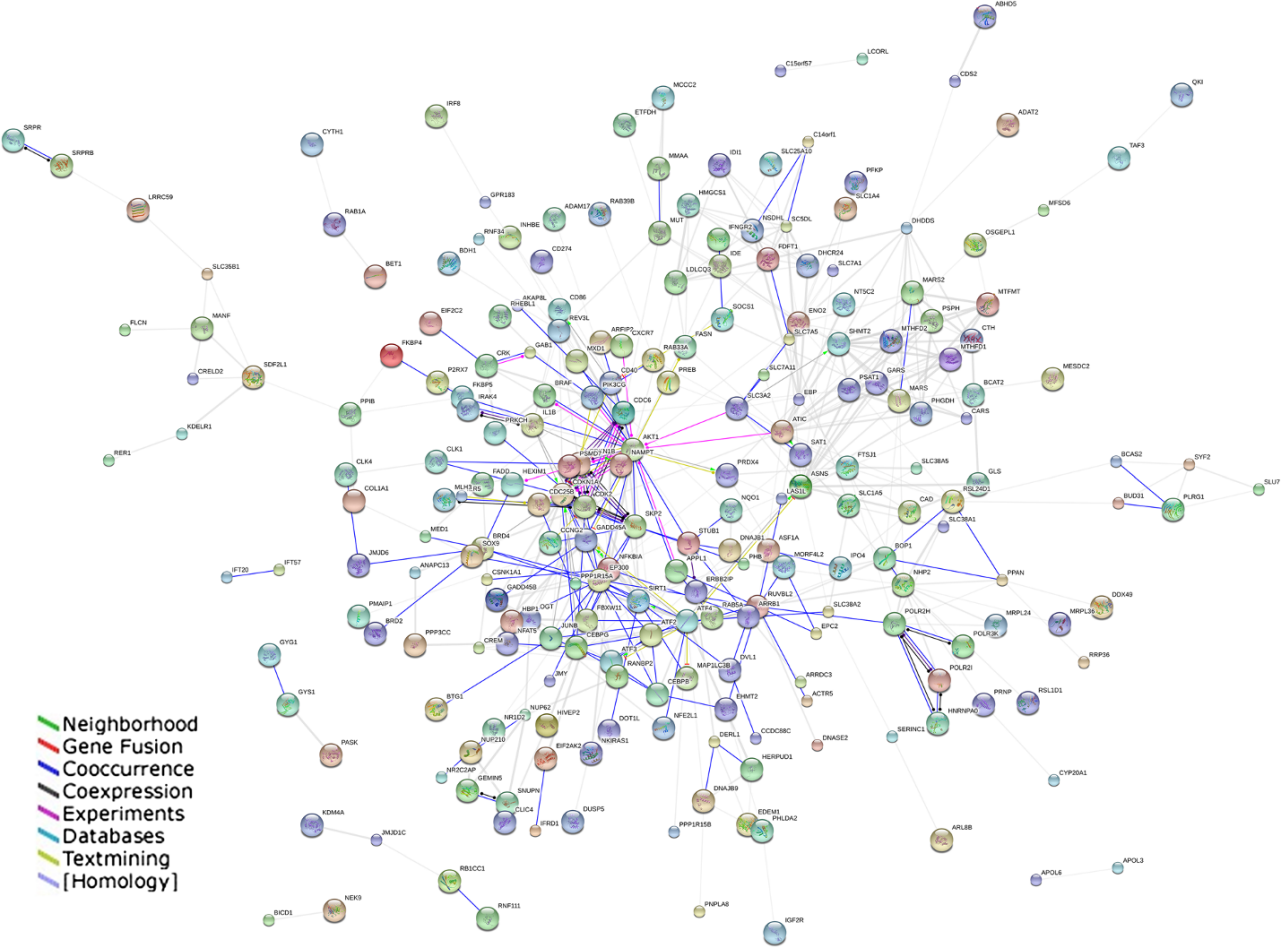
**Sub-networks constructed from differentially expressed PPI pairs.**

According to the HPRD database, there were a total of 36,875 PPI pairs. The differentially expressed genes were then projected to the PPI pairs, and we ensured that only the pairs in which the two interacting proteins were both differentially expressed were retained. Eventually, we obtained 375 differentially expressed relationship pairs. We used the Cytoscape software to build the sub-network diagram, and the results are shown in Figure 1.

**Figure 2:**
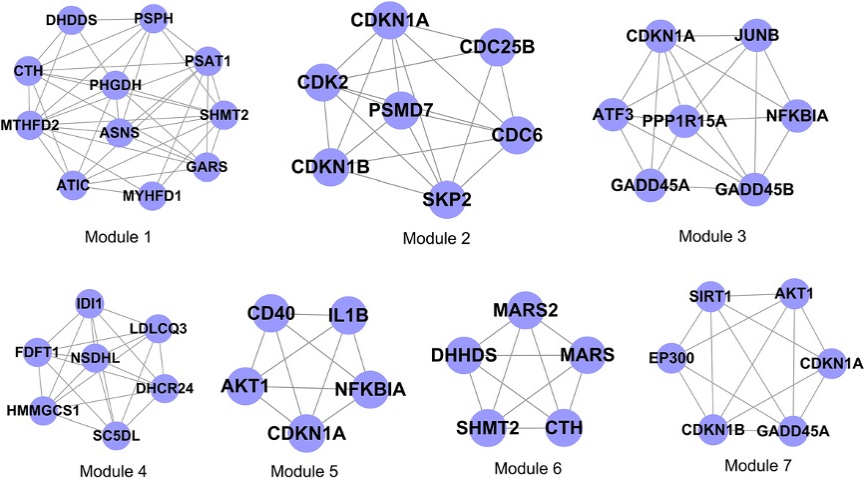
**Modular analysis results using CFinder software (k = 3).**

The present study used the CFinder software to conduct module identification for the differentially expressed PPI pairs obtained from the steps described above. When selecting k = 3, a total of 7 modules was obtained, as shown in Figure 2.

**Figure 3:**
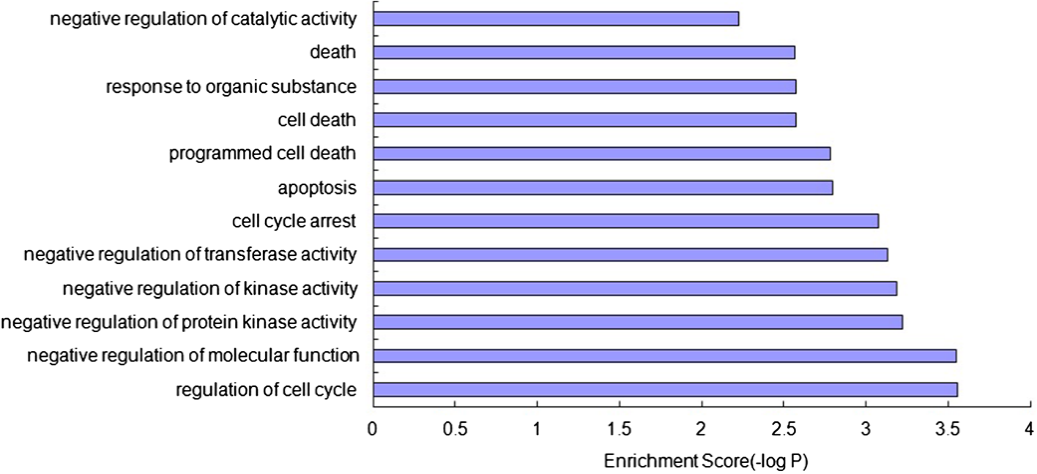
**GO-BP functional enrichment analyses of genes in module 3.**

We used the DAVID software to perform GO-BP functional enrichment analysis for each of the 7 modules. The functional analysis results showed that the functions enriched in module 3 were primarily related to "cell death" (Figure 3). We thus chose module 3 as the module of interest for further analysis.

**Figure 4:**
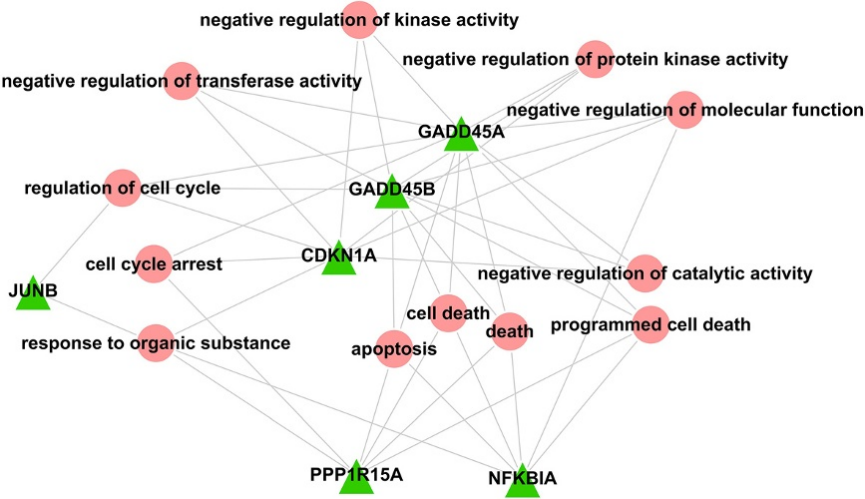
**Network diagrams of genes in module 3 and the corresponding GO-BP term.** Ellipse shape nodes represent GO-BP terms, and triangle shape nodes indicate genes.

There seven genes within module 3, including *NFKBIA*, *GADD45A*, *GADD45B*, *CDKN1A*, *JUNB*, *PPP1R15A* and *ATF3*. We constructed the relationship diagram for each gene and the corresponding GO-BP term using the Cytoscape software (Figure 4). The results indicated that *GADD45A*, *GADD45B*, *PPP1R15A* and *NFKBIA* were strongly related to "cell death".

### Construction and analysis of gene-drug networks

**Figure 5:**
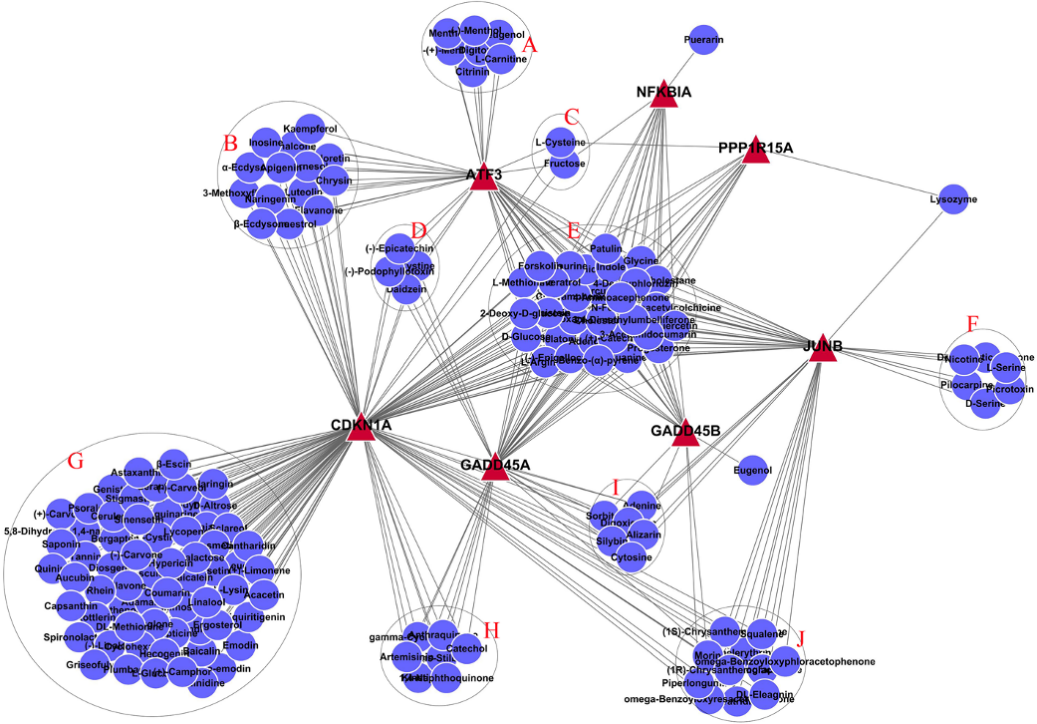
**Network diagrams of the relationships between herbal monomers and genes in module 3.** Ellipse shape nodes indicate herbal monomers, and triangle shape nodes indicate genes.

The gene-drug network diagram of the seven genes in module 3 and the herbal monomer library were constructed by literature mining (Figure 5). These results showed that the effects of some herbal monomers were multitargeted. Among them, 30 herbal monomers in group E can act simultaneously on the 7 genes in module 3, which were selected as the focus of our study.

## Discussion

Programmed cell death is a spontaneous cell death process committed to by cells with damage that cannot be repaired, in order to maintain homeostasis. There are three main types of programmed cell death: apoptosis, autophagy and necrosis [3]. Autophagy itself is a mechanism by which abnormal intracellular components are degraded to achieve homeostasis [3]. However, while presenting increased autophagic activity, cell death induced by many types of stress also shows morphological characteristics that do not fully comply with apoptosis or necrosis. Therefore, autophagy is considered a distinct mechanism of cell death [9, 10]. Autophagy plays an extremely important and complicated role in the pathogenesis and development of tumors. Studies have confirmed that autophagy is lower in tumor cells compared with normal cells in the same tissue during the early phase of tumorigenesis. Some tumors, such as liver cancer caused by chemical carcinogens, already demonstrate reduced autophagic capacity during the early phase of tumorigenesis. However, some other cancers (such as pancreatic cancer) showed increased autophagy during tumorigenesis [16]. After tumor induction by carcinogens, rat pancreas cells demonstrated normal autophagic capacity in precancerous nodules and adenomas, whereas this level of autophagy was reduced upon the formation of cancer cells [17]. Autophagy can first remove organelles (primarily mitochondria) damaged by chemical carcinogens, radiation and oxidative stress, thus avoiding genetic instability due to DNA damage caused by reactive oxygen species (ROS) and reducing the incidence of tumors. In addition, autophagy can degrade the endoplasmic reticulum, Golgi apparatus and other organelles, as well as proteins with long half-lives. Therefore, pre-malignant cells are in a negative balance of protein, which inhibits uncontrolled proliferation [18]. Indeed, excessive autophagic cell death can kill tumor cells, indicating the anti-cancer effect of autophagy. For example, tamoxifen can induce autophagic cell death in MCF-7 cells [19], and arsenic trioxide can stimulate autophagy in lymphocytic leukaemia and multiple myeloma cells [20]. After irradiation, Bax^-/-^Bak^-/-^ mouse embryonic fibroblast (MEF) cells showed reduced Akt/mTOR signalling and increased expression of ATG5, ATG12, Beclin1 and other autophagic factors; moreover, after Rad001 (mTOR inhibitor) treatment, autophagy was further enhanced, and MEF cells presented autophagic cell death [21]. Therefore, studies focusing on the regulatory mechanisms of autophagy and effective interventions for regulating autophagy have become an active area of cancer treatment research.

In this study, we focused on autophagy regulation and used microarray results from the GEO database to perform modular bioinformatics analysis using the CFinder, DAVID and Cytoscape software. We screened a gene group (module 3) composed of seven closely linked genes among the differentially expressed genes after the induction of autophagy. The functions of this gene group were primarily enriched in "cell death". In particular, four genes, *GADD45A*, *GADD45B*, *PPP1R15A* and *NFKBIA*, were directly related to "cell death".

*NFKBIA* (IkBα) encodes an inhibitor of NF-κB that regulates the activity of NF-κB by binding to and dissociating from NF-κB [22]. Studies have shown that in acute pancreatitis [23], the NF-κB signaling pathway regulates the expression of microtubule-associated protein light chain 3 (LC3) and Beclin-l to regulate the occurrence of pancreatic autophagy in rat acute pancreatitis. Genetic changes in *NFKBIA* are involved in colorectal [24] and glioblastoma tumorigenesis [25].

The GADD45 family consists of three genes (*GADD45A*, *GADD45B* and *GADD45G*) and have been shown to be involved in tumorigenesis [26]. The common feature of the GADD45 family is that the gene products can bind to the N-terminal domain of MTK1 and activate the MTK1 kinase and p38/JNK both *in vivo* and *in vitro*. It has also been shown that p38 plays a decisive role in the regulation of autophagy, and that SB203580 (a p38 specific inhibitor) can block the autophagy process [27]. Recent studies have shown that JNK participates in CH11 (Fas agonistic antibody)-induced autophagy, and further experiments have shown that the JNK inhibitor SP600125 can hinder the process of autophagy [28], suggesting that JNK and p38 are closely related to cellular autophagy. It can therefore be inferred that the GADD45 family of genes are closely associated with autophagy.

*CDKN1A*, also known as p21, participates in cell cycle regulation and inhibits or mediates apoptosis-related signaling pathways [29]. It has also been reported that in ceramide-treated MEFs, interference of p21 expression in p21^+/+^ MEFs led to a decreased level of apoptosis and increased autophagy, whereas p21 overexpression in p21^-/-^ MEFs enhanced autophagy and decreased the level of apoptosis [30]. *CDKN1A* has been shown to be associated with colorectal [31] and prostate cancer risk [32].

*JUNB* is also called Activator Protein 1 (AP-1) and is involved in skin [33] and breast cancer development [34]. Recent studies have found that in the process of autophagy, LC3 may bind specifically to the membrane structure of trans-Golgi network (TGN), and then TGN generates LC3-positive vesicles that bring LC3 to early autophagosome structures formed by the endoplasmic reticulum or other membrane structures [35]. In this process, the formation of AP-1 was shown to regulate clathrin vesicles together with ATG9 to regulate autophagy through the binding of LC3-PE to the corresponding complex and the formation of LC3-positive vesicles.

*PPP1R15A* (GADD34) dephosphorylates the eIF2α protein [36]. In MEFs, herpes simplex virus-induced PKR-dependent eIF2α phosphorylation was shown to induce autophagy. It has also been reported that eIF2α phosphorylation is necessary for amino acid deficiency-induced autophagy [37, 38]. Thus, eIF2α phosphorylation plays an important role in ER stress-induced autophagy.

From the above evidence, we can conclude that most of the genes in module 3 are closely associated with autophagy and that some of them are associated with cancer development. Because disease regulation by a single gene is not efficient, we introduced the concept of network pharmacology to examine how these genes are regulated, established a gene-drug network based on previously established disease-gene networks, and effectively combined these approaches to derive drugs for the treatment of diseases. The present study used the herbal monomer database to screen for monomers targeting the 7 genes in module 3. In particular, we performed literature mining of the PUBMED database and constructed the herbal monomer-module gene regulatory network using the Cytoscape software. We then selected 30 herbal monomers that could simultaneously act on these 7 genes and performed further literature searches. We found that herbal monomers such as resveratrol, curcumin, quercetin, and melatonin showed important relationships with autophagy.

Resveratrol is a polyphenol compound extracted from plants of the genus *Vitis*. Resveratrol possesses various biological functions, such as the induction of cell cycle arrest and apoptosis, as well as anti-tumor roles *in vivo*[39, 40]. Yamamoto et al. [41] showed that resveratrol could induce autophagy in glial cells; in addition, P38, MAPK and ERK1/2 MAPK participate in resveratrol-induced glial cell autophagy. Choi et al. [42] found that resveratrol treatment could induce protective autophagy in human dermal fibroblasts (HDFs) cultured under normal conditions. It has also been reported in the literature [43] that resveratrol can induce autophagy in different tumor cell lines including ovarian cancer [44, 45], colorectal cancer [46], gastric cancer [47], lung cancer and salivary gland cancer [48, 49]. Many pathways and mechanisms are involved in resveratrol-induced autophagy such as inhibition of the mTOR pathway and activation of the MAPK pathway [44, 50]. In addition, resveratrol-induced autophagy stimulates an accumulation of PELP-1 (proline-, glutamic acid-, and leucine-rich protein-1), which is a class of estrogen receptor co-activator in autophagosomes [49], as well as activation of Vps34 kinase and PI3K [46]. Together, these findings show that resveratrol can induce autophagy and may have an important role in tumor therapy. However, resveratrol is mainly associated with protective autophagy, and was identified in the present study because we studied autophagy genes that were influenced by herbal monomers.

Curcumin is a class of phenolic pigment extracted from turmeric in the genus *Curcuma* and is a natural phenolic antioxidant [51]. Turmeric roots contain a high content of curcumin. Numerous *in vivo* and *in vitro* experiments have shown that curcumin participates in the regulation of multiple cellular pathways, such as NF-κB, AKT, MAPK, p53, JAK/STAT and AMPK [52]. Related reports have demonstrated that curcumin can inhibit the growth of malignant glioma by inducing autophagy [53]. Curcumin-induced autophagy can also reduce the survival of cancer cells in hollow organs [54]. Li et al. [55] found that, in SKN cells, curcumin induced autophagy by activating the ERK1/2 pathway. In addition, treatment with an ERK1/2 inhibitor could reduce the ability of curcumin to induce autophagy and increase the ability of curcumin to induce apoptosis. Yamauchi et al. [56] reported that curcumin could increase LC3-II/LC3-I expression, induce the formation of autophagosomes and reduce ATG5 RNA silencing. Han et al. [57] found that curcumin prevented oxidative stress-induced endothelial cell damage by inducing autophagy. Together, these findings confirm that curcumin has an important role in the induction of autophagy.

Quercetin is a flavonoid antioxidant that is widely present in plants. Wang et al. [58] experimentally confirmed that quercetin could induce gastric cancer cells to form autophagic vacuoles, transform LC3-I into LC3-II, and activate autophagy genes. In addition, Kim et al. [59] found that quercetin could induce autophagy in U373MG cells, further confirming that quercetin initiates the autophagy process in gastric cancer cells.

Melatonin is an indole compound that has important physiological and pathological functions including regulating the body's circadian rhythms and seasonal rhythms. Choi et al. [60] found that in wild-type (WT) and GCD2-homozygous (HO) corneal fibroblast cell lines, melatonin could activate the initiation of autophagy. Recently, it was also reported that melatonin could prevent ischemia-reperfusion injury of the brain through the induction of autophagy [61]. Chen et al. [62] further demonstrated that melatonin could increase the LC3-II to LC3-I ratio and enhance autophagy. Overall, these findings show that melatonin plays an important role in the activation and initiation of autophagy.

In addition to the drugs mentioned above and shown to regulate autophagy, colchicine, patulin, chloramphenicol, genistein, L-arginine, benzo-(α)-pyrene and others have been reported to induce apoptosis and may therefore demonstrate regulatory potential for autophagy. However, the exact effects of all these monomers on autophagy and on the crosstalk between autophagy and apoptosis need to be confirmed *in vitro* and *in vivo*. In addition, autophagy may either promote cell survival or cell death, and the delicate balance between the two is ill understood [8]. The present study revealed possible effects of some monomers on genes involved in autophagy, but the results do not allow determining if these monomers would tip the balance one way or the other. Future studies are necessary to address these issues.

Colchicine is an alkaloid extracted from the plant *Colchicum autumnale* in the lily family [63]. Chen et al. [64] showed that colchicine could induce apoptosis through the mitochondrial pathway in L-02 cells. Patulin is a class of mycotoxin found in apples, grapes, pears and peaches, andt is an effective genotoxic compound. Saxena et al. [65] reported that patulin could induce DNA damage, resulting in p53-mediated cell cycle arrest and subsequent apoptosis. Kwon et al. [66] demonstrated that patulin acts via oxidative stress to induce phosphorylation of the transcription factor EGR-1 and thus increases the expression of ATF3, leading to cell cycle arrest and activation of the apoptotic protein cascade. Taurine, also known as β-aminoethanesulfonic acid, was first isolated from calculus bovis. Taurine is chemically stable and is a sulfur-containing non-protein amino acid that is closely associated with human metabolism. Zhang et al. [67] demonstrated that taurine could induce apoptosis in pulmonary artery smooth muscle cells via the death-receptor pathway. L-arginine is a semi-essential amino acid that has nutritional and immune regulation functions, can act as a precursor for the synthesis of nitric oxide, and is involved in various physiological and pathological processes in the body. Shu [68] found that L-arginine induces apoptosis by downregulating the expression and survival of the anti-apoptotic protein Bcl-2 and upregulating the expression of the pro-apoptotic protein p53. Genistein is an isoflavone found in soy and is a weak plant estrogen. Recent studies from Xia et al. [69] found that genistein could increase the expression of miR-34a, thereby inhibiting the expression of Notch-1 and promoting apoptosis. Kayisli et al. [70] demonstrated experimentally that genistein acts as an anti-proliferative factor and can stimulate human coronary artery endothelial cell apoptosis.

Therefore, some of the above-described herbal monomers deduced from the disease-gene-drug network have been shown to regulate the occurrence of autophagy, thus confirming the validity of this method. Some other herbal monomers deduced in this study have been used in the study of apoptosis but have not been reported to function in the regulation of autophagy, suggesting that we may find new drugs that can regulate autophagy among these herbal monomers.

The present study suffers from some limitations. Indeed, the aim of the present study was to use a new approach to screen for herbal monomers that could influence autophagy genes. However, each of these monomers will have to be carefully studied to determine their exact effects *in vitro* and *in vivo* and which way they normally tip autophagy (cell survival or death), and if the balance could be tipped the other way in specific circumstances. In addition, there is crosstalk between autophagy and apoptosis [9, 10], and future studies should evaluate the effects of different compounds on this crosstalk.

## Conclusion

This study provides a new strategy to discover herbal compounds for tumor treatment and disease alleviation that may assist with the identification of tumor cell autophagy inducers in the future.

## Authors’ information

Chenjun Hao is a medical doctor candidate, Zhengpeng Yang is a medical master candidate, Bo Gao is a medical doctor candidate, Xianzhi Meng is an associate professor, Dongbo Xue is a professor, and Weihui Zhang is a professor. The above authors are all from the Department of General Surgery, the First Affiliated Hospital of Harbin Medical University, Harbin, China.

Ming Lu is a assistant research fellow, Xin Qiao is a assistant research fellow, they both from Department of Surgery, David Geffen School of Medicine, University of California at Los Angeles, Los Angeles, CA, USA.
